# *Geranium robertianum* L. in Oral Health: From Phytochemical Profile to Therapeutic Potential

**DOI:** 10.3390/dj14080505

**Published:** 2026-08-10

**Authors:** Adina Feher, Larisa Bora, Diana Ungureanu (Similie), Corina Danciu, Ștefania Dinu, Ștefana Avram, Cristina Adriana Dehelean, Ramona Amina Popovici

**Affiliations:** 1 Doctoral School, “Victor Babeș” University of Medicine and Pharmacy Timișoara, Eftimie Murgu Square, No. 2, 300041 Timișoara, Romania; adina.feher@umft.ro (A.F.); diana.similie@umft.ro (D.U.); 2 Department of Management and Communication in Dental Medicine, Faculty of Dental Medicine, “Victor Babeș” University of Medicine and Pharmacy Timișoara, Eftimie Murgu Square, No. 2, 300041 Timișoara, Romania; ramona.popovici@umft.ro; 3 Research and Processing Center of Medicinal and Aromatic Plants, “Victor Babeș” University of Medicine and Pharmacy Timișoara, Eftimie Murgu Square, No. 2, 300041 Timișoara, Romania; corina.danciu@umft.ro (C.D.); stefana.avram@umft.ro (Ș.A.); 4 Department of Pharmacognosy-Phytotherapy, Faculty of Pharmacy, “Victor Babeș” University of Medicine and Pharmacy Timișoara, Eftimie Murgu Square, No. 2, 300041 Timișoara, Romania; 5 Department of Pedodontics, Faculty of Dental Medicine, “Victor Babeș” University of Medicine and Pharmacy Timișoara, Revolutiei Bv., No. 9, 300041 Timișoara, Romania; dinu.stefania@umft.ro; 6 Pediatric Dentistry Research Center, Faculty of Dental Medicine, “Victor Babeș” University of Medicine and Pharmacy Timișoara, Revolutiei Bv., No. 9, 300041 Timișoara, Romania; 7 Department of Toxicology, Drug Industry, Management and Legislation and Dermatopharmacy, Faculty of Pharmacy, “Victor Babeș” University of Medicine and Pharmacy Timișoara, Eftimie Murgu Square, No. 2, 300041 Timișoara, Romania; cadehelean@umft.ro; 8 Research Centre for Pharmaco-Toxicological Evaluation, “Victor Babeș” University of Medicine and Pharmacy Timișoara, Eftimie Murgu Square, No. 2, 300041 Timișoara, Romania

**Keywords:** *Geranium robertianum* L., phytochemical composition, dentistry, antimicrobial, antioxidant, periodontal disease, oral candidiasis, wound healing, oral cancer

## Abstract

**Background**: *Geranium robertianum* L. (GR) is a perennial herbaceous plant that belongs to the Geraniaceae family, widely distributed across temperate regions of Europe, Asia, North Africa, and America. It has been used in traditional ethnomedicine for its antibacterial, haemostatic, and anti-allergic properties in the treatment of oropharyngeal conditions, wounds, and inflammatory diseases. Beyond these traditional applications, pharmacological studies have documented several biological activities of its extracts and constituents. **Objective**: The aim of this review is to evaluate the therapeutic potential of GR by examining the phytochemical composition and the pharmacological properties relevant to dental medicine. **Methods**: A literature screening was conducted (PubMed, Google Scholar, PubMed Central), with focus on studies concerning phytochemical composition, pharmacological effects, and potential applications of GR and its major bioactive constituents in oral health. Results: Phytochemical analysis reveals that GR is characterized by a rich content of polyphenols, particularly tannins, flavonoids, and phenolic acids, with geraniin, ellagic acid, gallic acid, quercetin, and kaempferol identified as its principal bioactive constituents. Recent studies have confirmed that these constituents are responsible for the plant’s therapeutic potential in oral medicine. These compounds possess a variety of effects, from antibacterial and anti-inflammatory properties to antifungal and antiviral activities, as well as antioxidant, wound-healing, and anticancer benefits relevant to dental medicine. **Conclusions**: Despite its long-standing traditional use, GR remains largely underexplored in the context of oral health. This gap in the literature highlights GR as a promising phytotherapeutic candidate, calling for further in vitro and in vivo studies in order to investigate its efficacy and establish its mechanism of action in dental medicine.

## 1. Introduction

Oral diseases are among the most common health conditions globally, with an estimated 3.5 billion people affected. The World Health Organization’s (WHO) 2022 report on oral health provided a comprehensive overview of this burden, showing that oral diseases affect individuals across the entire life course, from early childhood to old age [1]. Four major problems stand out as the leading causes of these disorders: unaddressed tooth decay, serious gum infections, total loss of natural teeth, and oral cancers [1,2]. Far from being limited to just discomfort in the mouth area, such illnesses deeply affect overall physical well-being and daily living. They can cause issues like poor self-perception, retreat from social interaction, persistent pain, broken sleep patterns, mental strain, as well as difficulties carrying out basic functions, regardless of age [2].

Dental caries and periodontal diseases are among the most common preventable oral conditions. Their development is strongly linked to changes in the composition and balance of the oral microbiota [3]. Oral pathogens such as *Streptococcus mutans*, *Staphylococcus aureus*, and the opportunistic fungus *Candida albicans* often dominate diseased oral sites [4]. Current strategies in oral care commonly rely on synthetic agents such as fluoride, chlorhexidine, and triclosan. Nevertheless, these agents may cause side effects, including fluoride-related fluorosis in children and chlorhexidine-associated staining, dry mouth, taste alteration, and mucosal irritation. In this context, recent research has increasingly focused on natural compounds as complementary or alternative agents, mainly due to their broad biological activity and potentially better patient compliance [4,5]. Since antibiotics were introduced into clinical practice, their frequent and prolonged use has contributed to increasing antimicrobial resistance, pushing research toward new strategies that balance efficacy, safety, and reliability beyond conventional antimicrobial treatments [6].

Although synthetic drugs dominate modern dentistry, interest in plant-based treatments has grown steadily, mainly because of concerns related to side effects, antimicrobial resistance, and long-term tolerability [6]. In oral health, plant-derived extracts may offer several benefits, including antimicrobial, anti-inflammatory, antioxidant, analgesic, and wound-healing effects, which are relevant for conditions such as dental caries, periodontal disease, oral candidiasis, mucosal lesions, and toothache [3]. Flavonoids, along with certain tannins, have shown usefulness against oral infections and inflammatory processes. These compounds are now being explored in products such as toothpastes and mouthwashes, either as active ingredients or as supportive agents [6]. Whereas research into plant-based remedies is expanding rapidly, only a small proportion of potentially beneficial plant species has been thoroughly investigated, leaving considerable scope for future research [4].

GR, while still relatively underexplored, has recently attracted increasing scientific interest due to its complex phytochemical profile. Its extracts are rich in tannins, flavonoids, phenolic acids, and essential oil constituents, which have been associated with various health-related effects, including antioxidant, antimicrobial, anti-inflammatory, antidiabetic, neuroprotective, antiulcer, and anticancer activities [7,8]. In relation to oral health, GR aqueous extracts have shown bactericidal activity against *Streptococcus mutans* and *Streptococcus sobrinus*, two Gram-positive cariogenic bacteria. The activity reported mainly against Gram-positive strains suggests a potentially relevant antimicrobial profile for the control of cariogenic oral pathogens [8,9].

Even though early results appear encouraging, the precise mechanisms of action of GR are still unclear. More studies on its effects and medical uses remain essential in order to clearly elucidate the relationship between chemical structure and biological activity [8]. Up to now, there has been no overview focusing on its role in dental practice. To address this gap, the present review compiles the available research and evaluates the potential of *Geranium robertianum* L. in the prevention and treatment of oral diseases.

## 2. Materials and Methods

A literature search was conducted using PubMed, PubMed Central, and Google Scholar to identify relevant studies between 2005 and 2026 addressing the phytochemical profile, pharmacological activities, and potential dental applications of GR. The search was performed using the combinations of the following keywords: “*Geranium robertianum* L.”, “phytochemical composition”, “dentistry”, “dental medicine”, “oral medicine”, “antibacterial”, “antifungal”, “antiviral”, “antioxidant”, “periodontal disease”, “oral candidiasis”, “wound healing”, “oral cancer”. Reference lists of relevant publications were also manually screened to identify additional sources.

Following the initial research, the literature found was screened manually based on title, abstract, and full-text relevance. Eligible articles included original research papers, in vivo and in vitro experimental studies, and phytochemical characterization studies published in English. Studies focusing particularly on GR were preferentially selected. Additional studies involving other *Geranium* species or isolated phytoconstituents were included when they provided relevant support. Also, one PhD thesis meeting the established relevance criteria was included.

Exclusion criteria covered duplicate records, studies lacking direct relevance to the pharmacological and phytochemical profile of GR, and non- English publications. A total of 98 references were cited in the review, including 33 publications specifically addressing GR, 29 studies concerning its major bioactive constituents, 2 studies involving other *Geranium* species, 30 references providing background and contextual information, 1 PhD thesis, and 3 authoritative sources. The final literature search was updated on 27 July 2026. The selected literature was classified and organized according to botanical characterization, phytochemical composition, and pharmacological activities linked to oral medicine.

## 3. Botanical Description and Traditional Uses of *Geranium robertianum* L.

The Geraniaceae family comprises over 800 species [10]. Due to their volatile oil content, these plants are commonly used for ornamental purposes, as well as in aromatherapy, perfumery, and cosmetics [7]. The Geraniaceae family could be divided into 11 genera [11]. The genus *Geranium*, the main genus of the family, comprising approximately 400 species, is native to Central and Meridian Europe, as well as Asia [12,13]. The name of this plant genus is derived from the Greek term “géranos”, which means crane. This etymology reflects the appearance of the fruits, which are similar to the beak of a crane [10]. *Geranium* species have many established therapeutic uses, such as antiallergic, antiasthmatic, hemostatic, tonic, and antioxidant [12]. A taxonomic classification of GR is presented in Table 1.

GR, commonly referred to as Herb Robert, Red Robin, crow’s foot, or fox geranium, is an annual or biennial herbaceous species that arises spontaneously in fir, spruce, or beech forests, waste lands, or roadsides, typically at elevations exceeding 1500 m [12,15,16,17,18]. This plant thrives in highly diverse geographical regions, mainly temperate regions of the Northern Hemisphere, including Europe, North America, Asia, and North Africa [10,19,20]. It typically grows to a height of 10 to 60 cm and features long, thin, and branched stems that range from pale green to reddish colour. The leaves have approximately 10 cm in diameter, being triangular in shape, pinnate with three segments. The flowers are usually found in clusters of 2 to 4, are pink or purple, with a corolla made up of five rounded petals. The fruits resemble the beak of a crane [7].

Throughout history and into the present, GR has been recognized as a genuine medicinal plant, being used in different traditional medicine systems (e.g., Ayurveda, Chinese medicine) [10]. It has been used in various forms (e.g., infusion, decoction, maceration, mouthwash, gargles, liniment, or poultice) [7,21]. In folk medicine, GR was employed in the treatment of various human and animal conditions due to its antioxidant, antiallergic, antibacterial, anticancer, antidiarrhoeic, antihepatotoxic, stomachic, hemostatic, diuretic, and tonic properties [9,15,22]. Its traditional uses include treating ailments in numerous areas: ORL (tonsilitis, oropharyngeal inflammation, epistaxis, toothache, aphthous stomatitis, cough), infectious (labial herpes, grippe, scalp parasitosis), gastrointestinal (gallstones, ulcers, gastritis, diarrhea, jaundice), dermatological (wounds, burns, mild rashes), genitourinary (dispersal of kidney, leukorrhea, male infertility), metabolic (diabetes, hypercholesterolemia, gout), inflammatory diseases (arthritis, asthma, allergy), cancer [7,11,19,21,23,24,25,26,27,28,29,30]. The infusion or decoction of GR was employed as a hemostatic, antidiarrheic, coolant, and pain reliever. The GR tea was considered beneficial for stimulating lactation [13]. The infusion of GR flowers was employed for headaches, liver and stomach disorders, while infusion or decoction from GR leaves could be used for inflammations, diarrhea, hyperglycemia, or rheumatic pain [31,32]. More specifically, in traditional Peruvian medicine, both decoction and aqueous extract derived from GR bark are utilized in the treatment of various illnesses, including arthritis, gastritis, and cancer [12]. Within the Portuguese folk medicine, the infusions and decoctions from GR leaves were administered in order to normalize glycemia [33]. In northern Italy, in the southern Alps, infusions of GR flower and leaves are used in the treatment of fertility issues [34]. In Serbia and the Balkan Peninsula, GR has traditionally been consumed in order to treat various gastrointestinal and urinary conditions, while its topical application has been regarded for the treatment of wounds and mild rashes [35]. This plant is currently used in veterinary medicine, particularly the decoction from the whole plant, to treat scab in sheep, cattle, and horses [27]. Moreover, nowadays, herbal medicines based on *Geranium* are utilized in the treatment of some psychiatric disorders, including anxiety or depression [22]. There are also dietary supplements containing GR extracts, which are recommended for fitness purposes, including weight loss [31]. Moving forward, multiple studies have demonstrated the cytotoxic effect of GR against several cancer cell lines, including MCF-7, HeLa, HepG2, and NCI-H460 [9]. Additionally, some studies have indicated that GR can also function as a cytostatic agent against malignant cells [31].

## 4. Phytochemical Composition

**Figure 1:**
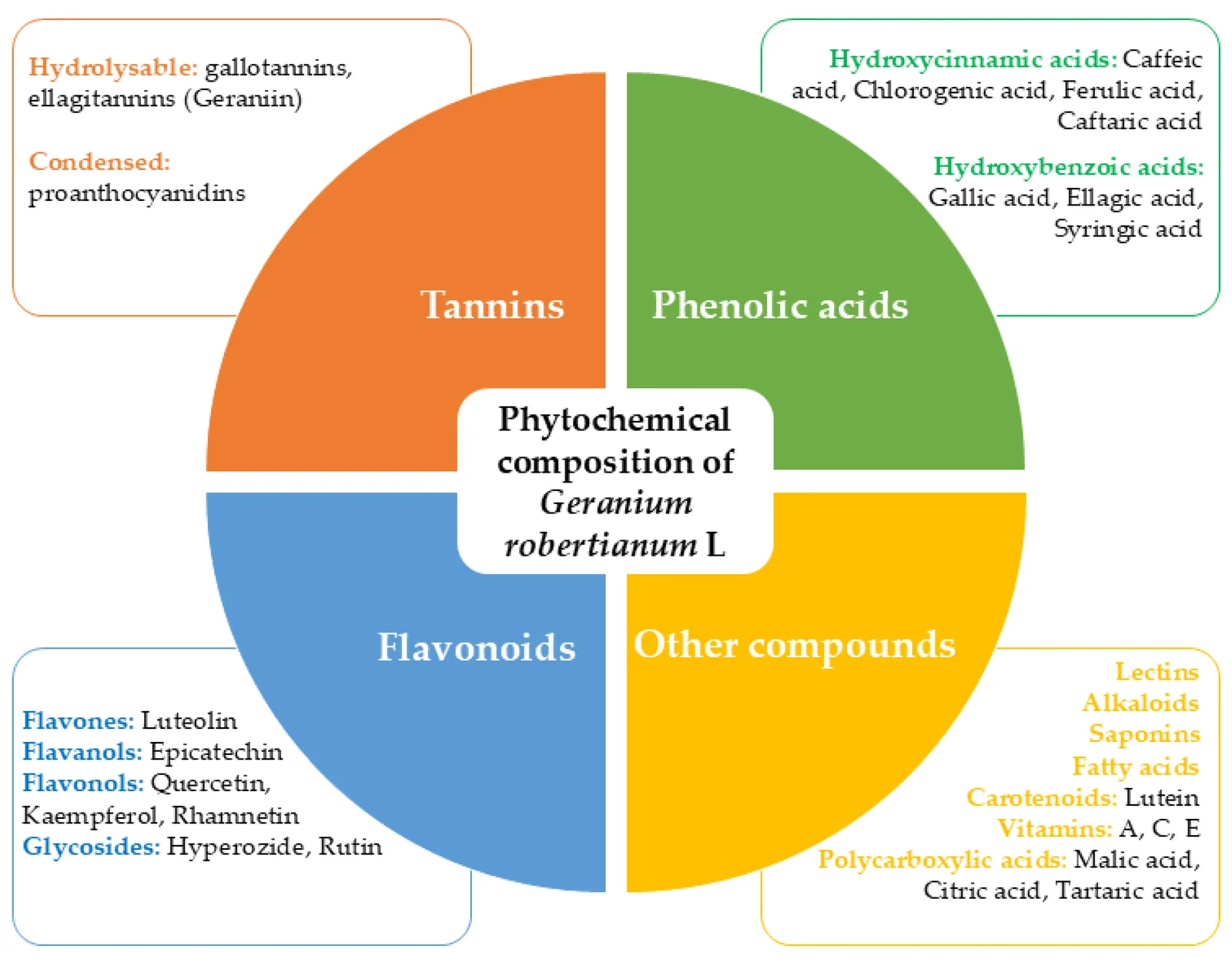
Phytochemical composition of GR.

The pedoclimatic conditions have a substantial impact on the phytochemical composition of plants. GR is a versatile plant that can thrive in both non-polluted and polluted soils without significant accumulation of heavy metals [36]. Furthermore, GR is widely recognized for its capacity to absorb minerals from the soil [7]. It was observed that extracts from Romanian GR contain significant amounts of Ca, Mg, and Fe, while Pb was found to be below the detection limit, indicating the plant’s safety [37]. Regarding the nutritional profile of GR, it was observed that the main macronutrients present in this plant are represented by carbohydrates (over 50 g/100 g dry weight), followed by proteins, fat, and ash [26]. The principal bioactive compounds present in the phytochemical composition of the *Geranium* genus comprise polyphenols, particularly tannins, flavonoids, and phenolic acids [10,15]. The plant products of GR are known for their various content of secondary metabolites, including tannins, flavonoids, polyphenolic acids, saponins, alkaloids, and other compounds (Figure 1) [12]. The significant phenolic content is highly correlated to the antioxidant, anti-inflammatory, antimicrobial, and cytotoxic potential of GR extracts [10].

Table 2 displays the total phenolic content, total flavonoid content, and the quantities of specific compounds found in various GR extracts.

The phytochemical profile of GR extracts highlights the presence of a variety of secondary metabolites, including significant amounts of flavonoids and phenolic compounds [15]. Table 2 summarizes the total phenolic content of polyphenols and flavonoids, as well as the main phytochemicals identified in the GR extracts described in the literature. Additionally, the information presented in this table points out the diversity of experimental approaches employed to characterize the phytochemical profile of GR species. Regarding polyphenols, chlorogenic acid and caffeic acid, belonging to hydroxycinnamic acids, are present in the greatest amounts, along with hydroxybenzoic acids such as gallic and ellagic acids. Among the flavonoids, rutin, quercetin, and luteolin are the most abundant. These classes of phytocompounds are well recognized for their antioxidant and anti-inflammatory properties, as well as for their contribution to the biological activity of the plant extracts [20]. Multiple clinical trials have demonstrated the therapeutic benefits of flavonoid-rich plant extracts or supplements in lowering oxidative stress and inflammation, as well as improving the overall health and quality of life [44,45].

The leaves and stems of GR are also very abundant in tannins. Ellagitannins and ellagic acid are the major phenolic compounds found in the decoctions of leaves and stems, accounting for approximately 50% of the total phenolic content [31]. Ellagitannins and gallotannins are the main types of hydrolysable tannins, derived from ellagic and gallic acids [46]. Ellagitannin geraniin could be extracted from all types of leaves in the *Geranium* species [25]. Due to their astringent activity, these compounds possess significant potential in the treatment of diarrhea as well as in cases of poisoning with heavy metals or alkaloids [21]. Moreover, this tannin is recognized for several other pharmacological properties, including anti-cancer, antiviral, anti-hypertensive, and anti-hyperglycemic [47]. Geraniin exhibits significant antiviral properties against influenza A and B, herpes simplex virus (HSV), and human immunodeficiency virus 1 (HIV-1). This compound holds potential for application in both the treatment and prevention of severe acute respiratory syndrome coronavirus 2 (SARS-CoV-2) infection [48]. Ellagic acid, which is related to geraniin, and its precursor, gallic acid, are mainly distributed in the plant roots [21]. Ellagic acid also possesses antiviral potential against HIV-1, influenza A, Zika, Ebola, hepatitis B virus, and rhinoviruses [49]. The infusion of aerial parts was found to contain carotenoids such as lutein [7].

The essential oil of GR aerial parts is rich in fatty acids. Its significant antimicrobial properties are due to the high content of monoterpenoids with a hydroxyl group (geraniol, linalool, α-terpineol), monoterpenes (γ-terpinene), but also sesquiterpenes (β-caryophyllene) [50]. The significant amounts of geraniol and citronellol, phytocompounds that possess impressive antifungal properties against fungi species, make GR essential oil a great candidate against *Candida albicans* [51]. There were observed differences in the phytocomposition of the essential oil extracted from the aerial parts and underground parts of GR (Southeast Serbia). The oil from the aerial parts was found to be richer in terpenoids (26.7% versus 1.5%), while the oil from the underground parts possessed higher amounts of fatty acids and derivatives (93.4% versus 49.2%). Hexadecanoic acid, pentacosane, and hexahydrofarnesyl acetone were found to be the most abundant phytocompounds in the GR oils [17]. Besides this, the essential oil obtained by hydrodistillation from the aerial parts of GR (Trabzon, Turkey) was found to contain significant amounts of (E)-caryophyllene (11.3%), α-fenchylacetate (11.2%), and limonene (9.7%) [52].

## 5. Therapeutic Actions Relevant to Dentistry

### 5.1. Antibacterial Activity in Dental Caries

Dental caries remains one of the most widespread oral health challenges worldwide, with *Streptococcus mutans* recognized as a key bacterial species involved in its development. This microorganism thrives in dental plaque, the biofilm that adheres to tooth surfaces, where it contributes to enamel demineralization. Its ability to adapt rapidly to fluctuating conditions within dental plaque, including changes in pH, nutrient availability, and microbial competition, is central to its cariogenic potential. Consequently, increasing attention has been directed toward strategies able to limit cariogenic biofilm formation by targeting *S. mutans*, while preserving the balance of the healthy oral microbiome [53].

In this context, plant-derived compounds have emerged as interesting candidates due to their multi-target antibacterial mechanisms and lower likelihood of inducing bacterial resistance [4]. Polyphenols, particularly flavonoids and tannins, are especially relevant in dental caries because they may interfere with bacterial adhesion, biofilm formation, membrane integrity, energy metabolism, and key enzymes involved in virulence, such as glucosyltransferases and sortase A [4,6]. Tannins and related polyphenolic compounds may also alter the bacterial cell membrane and disturb the redox balance of *S. mutans*, leading to metabolic stress and reduced bacterial viability [4,54].

With respect to the direct antibacterial activity of GR, several types of extracts, including methanolic, aqueous, ethanolic, and hexane extracts, have shown remarkable activity against both Gram-positive (*Staphylococcus aureus*, *Streptococcus* spp., *Bacillus cereus*) and Gram-negative bacteria (*Escherichia coli*, *Salmonella infantis*), as well as against fungal strains such as *Candida* spp. [43]. Świątek et al. investigated extracts obtained from the aerial parts of GR (commercially sourced from Poland) against a panel of Gram-positive and Gram-negative bacterial strains, including species of dental relevance such as *Staphylococcus aureus*, *Staphylococcus epidermidis*, and *Enterococcus faecalis*. Overall, susceptibility to GR extracts was observed among Gram-positive bacteria, whereas Gram-negative strains were weakly affected or unaffected. Among the tested extracts, the GR hexane extract showed the strongest inhibitory activity against Gram-positive strains, with minimum inhibitory concentration (MIC) values ranging from 0.06 to 0.5 mg/mL, followed by the ethyl acetate extract, with MIC values ranging from 0.03 to 1 mg/mL. The methanolic extract expressed weaker but still relevant activity, with MIC values ranging from 0.06 to 2 mg/mL against Gram-positive bacteria. Notably, the aqueous extract displayed bactericidal effects against all Gram-positive bacteria tested, as reflected by minimum bactericidal concentration (MBC)/MIC ratios ranging between 1 and 4 [9]. Another study assessed the antimicrobial potential of three types of GR extracts, namely methanolic, dichloromethane, and crude aqueous extracts obtained from different plant parts. The dichloromethane extract from GR leaves significantly inhibited the growth of *S. aureus*, with an inhibition zone of 8 mm at 1 mg/mL [8].

Of particular interest for dental caries, the aqueous whole-plant extract of GR demonstrated bactericidal effect against *Streptococcus mutans* and *Streptococcus sobrinus*, two closely related cariogenic bacteria [8,9]. Furthermore, the ethanolic extract of GR was tested against five reference bacterial strains (*Streptococcus mutans* ATCC 35668, Streptococcus pyogenes ATCC 19615, *Staphylococcus aureus* ATCC 25923, *Escherichia coli* ATCC 25922, *Pseudomonas aeruginosa* ATCC 27853), and showed a moderate inhibitory effect, with inhibition zones generally similar to that of levofloxacin [43].

**Figure 2:**
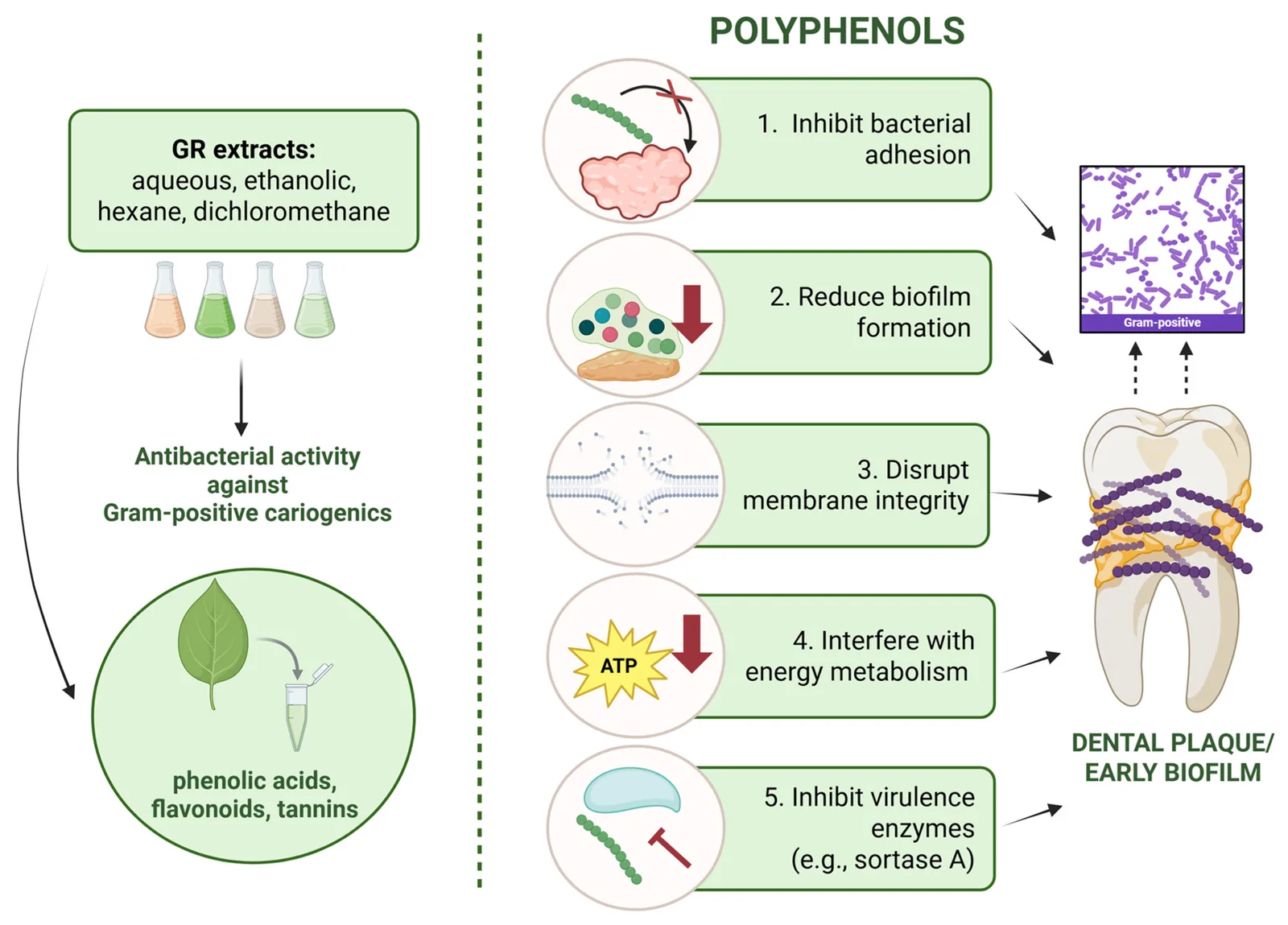
Antibacterial activity of GR and mechanisms reported for bioactive compounds also identified in the plant, relevant to dental caries. Created with BioRender.com.

Concluding, the available evidence indicates that GR may represent a promising phytotherapeutic candidate for the prevention of dental caries, particularly due to its activity against Gram-positive cariogenic bacteria (Figure 2) [9]. Nevertheless, most studies remain limited to in vitro models, which do not fully reproduce the complexity of the oral environment, including saliva, biofilm architecture, microbial interactions, pH variations, and the stability of natural compounds under oral conditions [9]. Moreover, it remains unclear whether GR extracts selectively reduce cariogenic species such as *S. mutans* while preserving the commensal oral microbiota [53].

### 5.2. Anti-Inflammatory and Antibacterial Activity in Periodontal Disease

Periodontal disease is a degenerative inflammatory disease involving gingivitis, periodontitis, pericoronitis, and peri-implantitis. It is caused by dysbiotic subgingival microbiota and dysregulated host immunological response. The pathogenesis involves the release of pro-inflammatory cytokines (interleukin-1 beta (IL-1β), interleukin-6 (IL-6), tumoral necrosis factor-alpha (TNF-α), prostaglandin E2 (PGE2)) and matrix metalloproteinases (matrix metalloproteinase-8 (MMP-8), matrix metalloproteinase-9 (MMP-9)) that work together to break down collagen and the extracellular matrix, resulting in alveolar bone resorption and tooth loss [6,55]. *Porphyromonas gingivalis* is regarded as the principal periodontal pathogen, and its lipopolysaccharide (LPS) is the principal driver of local inflammation [56]. Periodontal disease represents a major therapeutic challenge affecting over 50% of adults worldwide, and adjuvant herbal medications are being extensively researched [55]. GR represents a promising candidate in this regard, as the main phytochemicals, namely ellagitannins, ellagic acid, gallic acid, quercetin, and oligomeric proanthocyanidins, have been described to act separately on the same molecular pathways involved in the pathogenesis of periodontal disease [8].

Direct evidence of the anti-inflammatory effects of GR has been shown by several studies. The 50% hydroalcoholic extract, commercially purchased, was found to be moderately inhibiting hypochlorous acid-mediated oxidation, the principal oxidizing agent generated by neutrophils in acute inflammation [8]. An aqueous extract of GR aerial parts (obtained from Warsaw, Poland) inhibited the activity of elastase, an enzyme that is crucial in the destruction of the extracellular matrix, by 34.7% at 10 µg/mL [41]. Also, Catarino et al. demonstrated that aqueous decoctions of GR leaves and stems (collected from wild flora in Cernache do Bonjardim, Portugal) neutralized the nitric oxide radical (NO•), a major mediator of inflammation, with half maximal inhibitory concentration (IC_50_) values of 20.0 ± 0.9 µg/mL and 24.2 ± 8.0 µg/mL, respectively, which were about ten times more potent than ascorbic acid. The stem extract also revealed a dose-dependent inhibitory effect on LPS-induced nitrite generation in RAW 264.7 macrophages [31].

Ellagitannins are the most abundant phytochemicals in GR [8,31]. Granica et al. demonstrated that ellagitannins modulate the inflammatory response of human neutrophils by mechanisms that involve the inhibition of elastase, reactive oxygen species (ROS), interleukin-8 (IL-8), TNF-α, and MMP-9 release, downregulation of toll-like receptor 4 (TLR-4) surface expression, and induction of neutrophil apoptosis, a mechanism of particular relevance in the resolution of chronic oral inflammation [57]. Additionally, ellagitannins are strong MMP inhibitors, as evidenced by Malcangi et al., who demonstrated that they decrease IL-1β and TNF-α production and hence limit the destruction of periodontal tissue [55].

Furthermore, in an experimental rat model of periodontitis, ellagic acid showed protective effects on periodontal tissues after systemic administration (15 mg/kg). Treatment was associated with lower alveolar bone resorption and reduced oxidative imbalance, together with a favourable inflammatory profile, reflected by decreased gingival IL-6 and TNF-α and increased interleukin-10 (IL-10) levels [58]. Polyphenols have been proven to inactivate bacterial toxins, increase the antioxidant activity of oral fluids, have an antibacterial effect on periodontal pathogens, and block the proteolytic activity of *P. gingivalis* [58].

Another phenolic acid present in GR is gallic acid, which was shown by Dai et al. to improve experimental periodontitis by suppression of LPS-induced nuclear factor kappa-light-chain-enhancer of activated B cells (NF-κB) phosphorylation and generation of IL-1β and TNF-α, and also deterioration of alveolar bone. The clinical relevance of gallic acid is further evidenced by the use of the gallic acid-based Xipayi mouth rinse, which has shown effectiveness against oral bacteria associated with gingivitis and mild periodontitis, leading to reduced plaque accumulation and gingival bleeding [59].

Xiong et al. investigated the flavonoid quercetin isolated from GR in human gingival fibroblasts activated by *P. gingivalis* LPS. The findings revealed that quercetin reduced the production of IL-1β, IL-6, IL-8, and TNF-α in a dose-dependent way. Also, it suppressed TLR4-mediated NF-κB activation and showed anti-inflammatory actions via peroxisome proliferator-activated receptor gamma (PPARγ) activation [56]. López-Valverde et al. confirmed these data by reporting that quercetin inhibits alveolar bone resorption and pro-inflammatory cytokines. In a systematic investigation of 14 natural products, the researchers identified the highest degree of evidence (+++) for antioxidant activity, and moderate evidence (++) for anti-inflammatory and antibacterial activity in periodontal disease [60].

**Figure 3:**
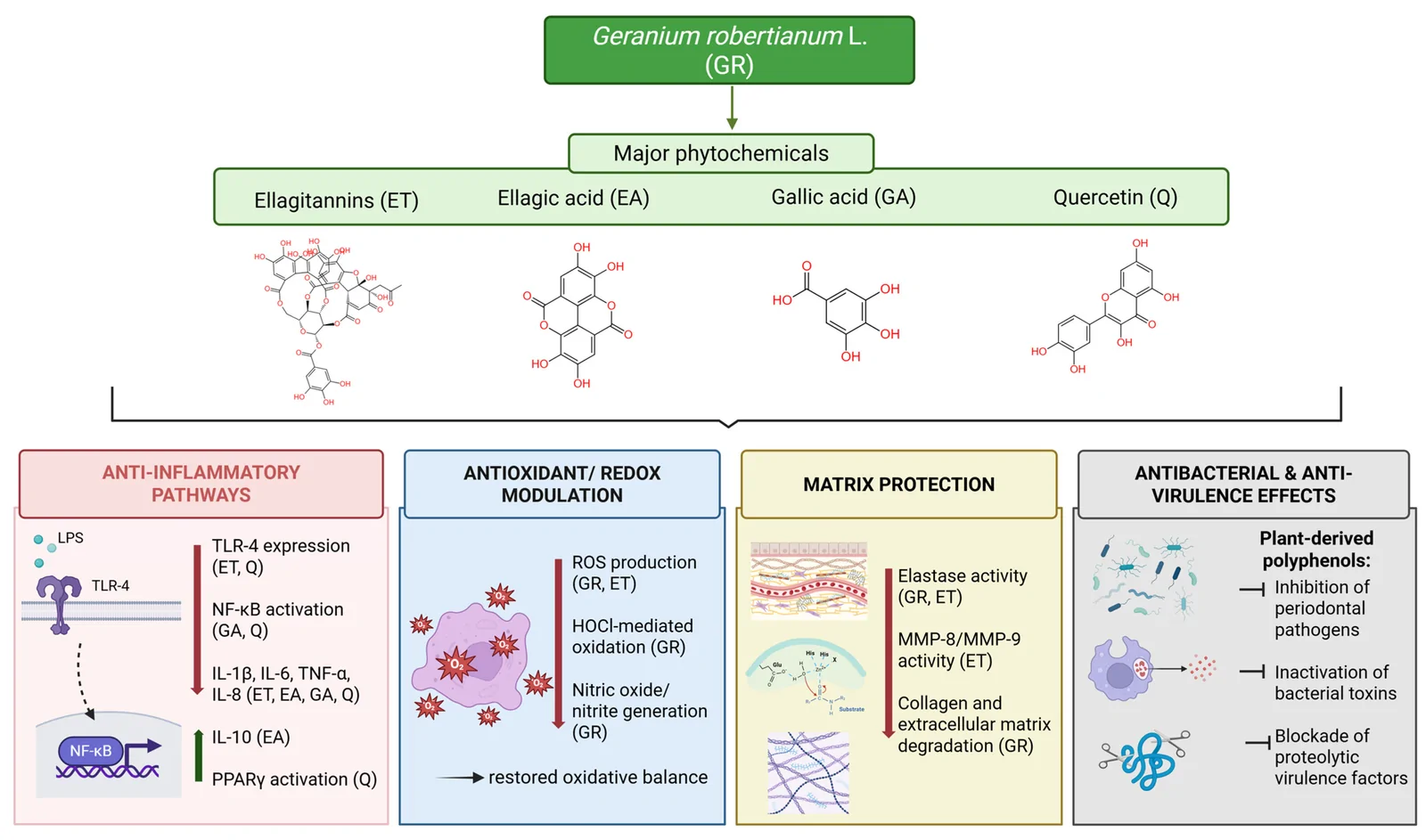
Anti-inflammatory and antibacterial mechanisms relevant to periodontal disease: evidence from GR and bioactive compounds also found in the plant. Created with BioRender.com.

Compared with the evidence available for GR extracts, studies on individual constituents provide findings that are more directly relevant to periodontal disease, including data from experimental periodontitis models and oral cell lines (Figure 3).

The phytochemical profile of GR suggests its potential relevance in periodontal disease, primarily due to the presence of ellagitannins, ellagic and gallic acids, and quercetin, compounds associated with anti-inflammatory and antioxidant effects. Although direct evidence regarding their effects as constituents of GR in periodontal disease remains limited, their reported ability to reduce oxidative stress, inflammatory mediator release, and matrix degradation suggests that they may influence mechanisms involved in periodontal tissue damage [6,55,60]. Further studies directly evaluating GR in periodontal disease models are therefore required.

### 5.3. Antifungal Effect in Oral Candidiasis

Oral candidiasis is most commonly caused by *Candida albicans*, which accounts for approximately 80% of infections, although non-albicans species such as *Candida glabrata*, *Candida tropicalis*, *Candida krusei*, and *Candida dubliniensis* are increasingly reported [61,62]. Environmental conditions can influence the expression of fungal virulence genes and thus affect the clinical outcome of infection [62]. Common antifungal agents include nystatin, fluconazole, and newer systemic injectable therapies. However, their use may be associated with side effects, poor targeting, increased tolerance, and limited effects on the inflammatory component of oral candidiasis [61]. Molecules found in GR, including flavonoids, tannins, phenolic acids, and essential oil constituents, show biological promise due to their antifungal and anti-inflammatory effects [8,9].

The antifungal activity of GR has been investigated using different types of extracts. Swiatek et al. evaluated extracts obtained from the whole GR plant (commercially purchased from Koryciny, Poland) and reported that the ethyl acetate extract and methanolic extract were active against several yeast strains, with MIC values ranging from 1 to 8 mg/mL. The ethyl acetate extract inhibited the growth of *Candida albicans* (ATCC 2091 and ATCC 10231 strains) at concentrations as low as 1 mg/mL. Other strains, such as *C. glabrata*, *C. parapsilosis*, *C. krusei*, and *C. tropicalis*, were sensitive to both ethyl acetate and methanolic GR extracts. Low minimum fungicidal concentration (MFC)/MIC ratios of 1–4 indicated fungicidal activity for the aqueous and hexane extracts, whereas *Candida auris* was resistant to all tested extracts [9]. Further, it was shown that methanolic extracts obtained from GR stems (provided from Koura, Lebanon) inhibited *C. albicans*, with an inhibition zone of 9 mm at 10 mg/mL [12]. Notably, the antifungal activity varied according to the extraction solvent, plant material, tested concentration, and *Candida* species.

In relation to oral candidiasis, several GR constituents may also be relevant. Ellagic acid showed antifungal and anti-inflammatory potential when formulated as a cyclodextrin complex (ellagic acid/hydroxypropyl beta-cyclodextrin (HP-β-CD) complex), reducing the invasive capacity of *C. albicans* [63]. Geraniol, a volatile compound present in geranium essential oils, suppressed tested *Candida* strains (MIC = 1.25–5 mM/mL) and quenched the inflammation markers IL-1β, IL-6, and interleukin-18 (IL-18), and the fungal virulence factors secreted aspartyl proteinase 1 (*SAP1*) and phospholipase B1 (*PLB1*) [64]. Kovac et al. emphasize that flavonoids and tannins, isolated from *Rubus idaeus* L., exert antifungal effects against the oral pathogen *C. albicans*, supporting their relevance in the management of oral infectious diseases [6].

**Figure 4:**
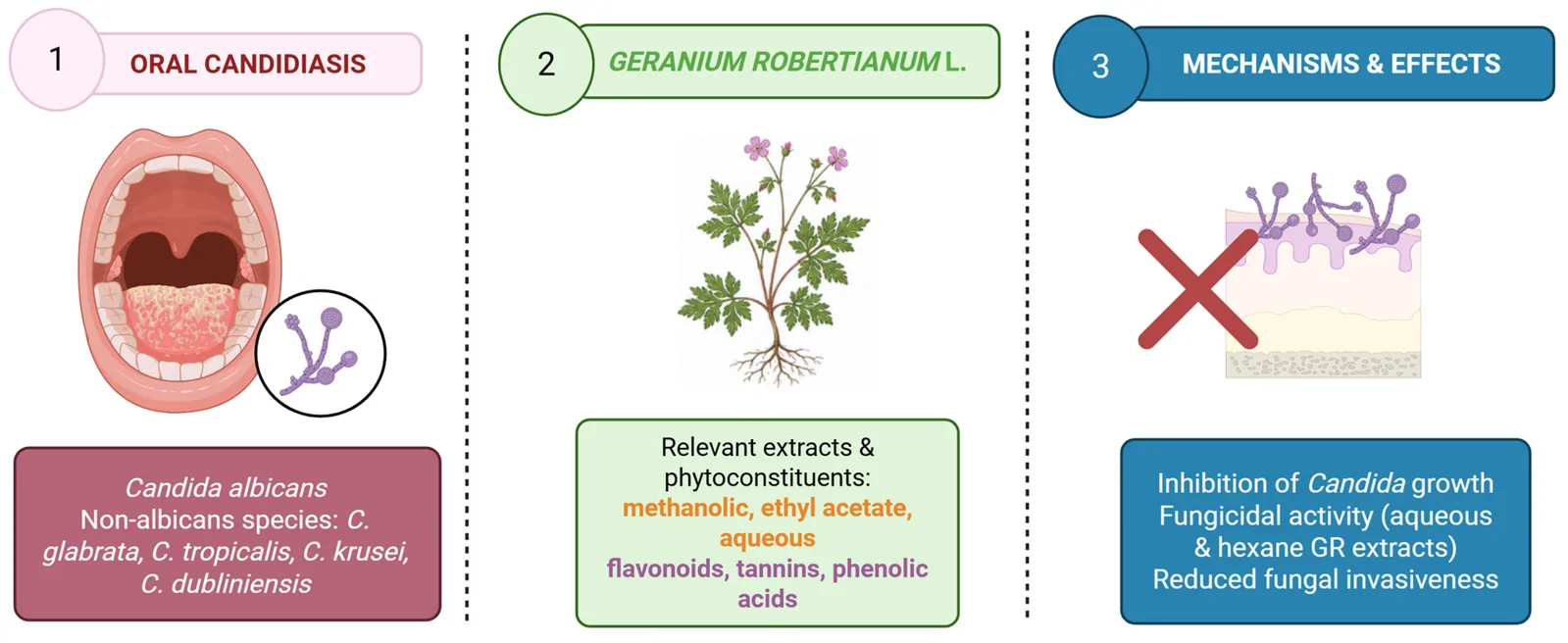
Antifungal mechanisms reported for GR and for bioactive constituents also found in the plant, relevant to oral candidiasis. Created with BioRender.com.

The available data indicate that different types of GR extracts, along with their bioactive molecules, show antifungal activity against *C. albicans* and several non-albicans *Candida* species [8,9]. Their potential relevance in dentistry is related not only to the inhibition of fungal growth but also to the reduction in fungal invasiveness, virulence factor expression, and local inflammatory responses (Figure 4) [6,63,64,65,66].

### 5.4. Antiviral Activity in Oral Viral Infections

HSV-1 infection is widespread globally, with the WHO estimating that approximately 3.8 billion people under 50 years are infected, namely 64% of the global population. As HSV-1 is the main cause of oral herpes, recurrent orolabial lesions remain clinically relevant in dental practice, particularly in immunocompromised individuals [67,68]. Although acyclovir and related antiviral agents remain the standard therapeutic options, their effects in lesion duration and pain relief are limited, and resistant viral strains may occur, particularly in vulnerable patients. Therefore, the investigation of plant-based remedies with antiviral potential is currently encouraged [68].

Geranium-derived geraniin shows antiviral activity against pathogens such as HIV, hepatitis B, and HSV. It has been determined that geraniin significantly reduced HSV-1 plaque formation at 8.3 µg/mL [69]. Other ellagitannins from plant sources, including castalagin, vescalagin, and grandinin, have also been reported to suppress acyclovir-resistant HSV replication, possibly through interference with viral cell entry [70,71].

Other bioactive molecules identified in GR further support its antiviral relevance. Ellagic acid showed stronger inhibition than ribavirin against three human rhinovirus (HRV) types, acting early on host cells rather than directly on the virus [72]. Further, quercetin reduced the frequency and duration of outbreaks when used alongside standard therapy in a study involving 68 patients with recurrent oral herpes compared to results registered under treatment with acyclovir or valacyclovir alone [73]. Kaempferol has also been associated with antiviral effects, including reduced hepatitis B viral DNA replication and inhibition of Epstein–Barr virus reactivation [74,75]. Thus, among the described findings, the studies on geraniin against HSV-1 and on quercetin in recurrent oral herpes provide the most relevant evidence regarding oral viral infections.

**Figure 5:**
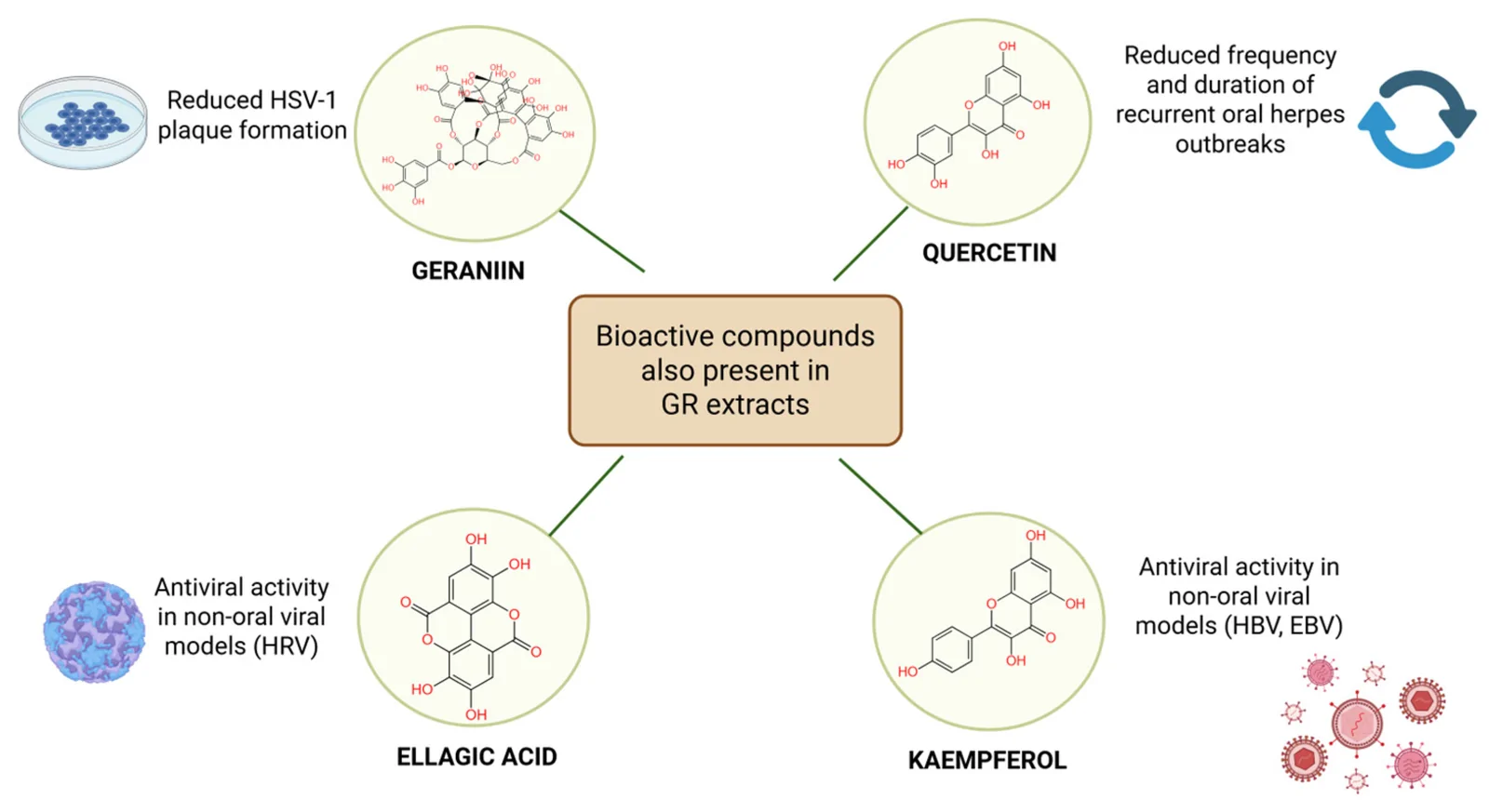
Reported antiviral mechanisms for bioactive constituents in oral viral infections. Created with BioRender.com.

Based on the reported activities of bioactive constituents also identified in GR (Figure 5), this plant could represent a source of bioactive molecules with potential adjunctive relevance in the management of recurrent oral viral lesions.

### 5.5. Antioxidant Activity

Oxidative stress plays an important role in several oral pathologies, including dental caries, periodontal disease, potentially malignant disorders, and oral cancer [76]. In periodontal tissues, excessive ROS can trigger inflammation, cellular damage, and activation of enzymes involved in extracellular matrix degradation [76,77]. In dental caries, oxidative imbalance has also been associated with reduced salivary antioxidant capacity and increased markers of cellular stress [78]. According to Liu and Guo, almost one in five adults worldwide suffers from advanced periodontal disease, whereas about half face milder versions across populations [79]. Because of this shortfall, antioxidant natural compounds are increasingly investigated as adjunctive agents in oral medicine, particularly for conditions associated with inflammation and redox imbalance [76,79].

GR contains several polyphenolic compounds with antioxidant potential, such as geraniin, ellagic acid, gallic acid, and caffeic acid, with high relevance for oral health [8]. According to Haj Ali et al., the antioxidant activity of GR extracts is closely correlated with the number of hydroxyl groups in the structure of the polyphenolic compounds, which can neutralize ROS such as hydroxyl radicals, hydrogen peroxide, and superoxide, thereby limiting lipid peroxidation and DNA damage [8]. Researchers highlighted that methanolic GR root extract (Koura, Lebanon) inhibited 97% of the 2,2-diphenyl-1-picrylhydrazyl (DPPH) radical at a concentration of 0.3 mg/mL, while the methanolic extract of GR leaves (collected from Hammam-Lif, Tunisia) showed antioxidant capacity in the β-carotene/linoleic acid bleaching assay, with an IC_50_ of 6.8 µg/mL, superior to butylhydroxytoluene (IC_50_ = 85 µg/mL) [12,15].

Geraniin further supports the antioxidant potential of GR. Ilić et al. reported that extracts obtained from the underground parts of GR, collected from Jabukovik and Sastav Reka, Serbia, showed strong antioxidant capacity, exceeding that of Trolox. Geraniin has also been associated with the modulation of oxidative stress markers and the improvement of antioxidant defence systems, including glutathione-related mechanisms [39].

**Figure 6:**
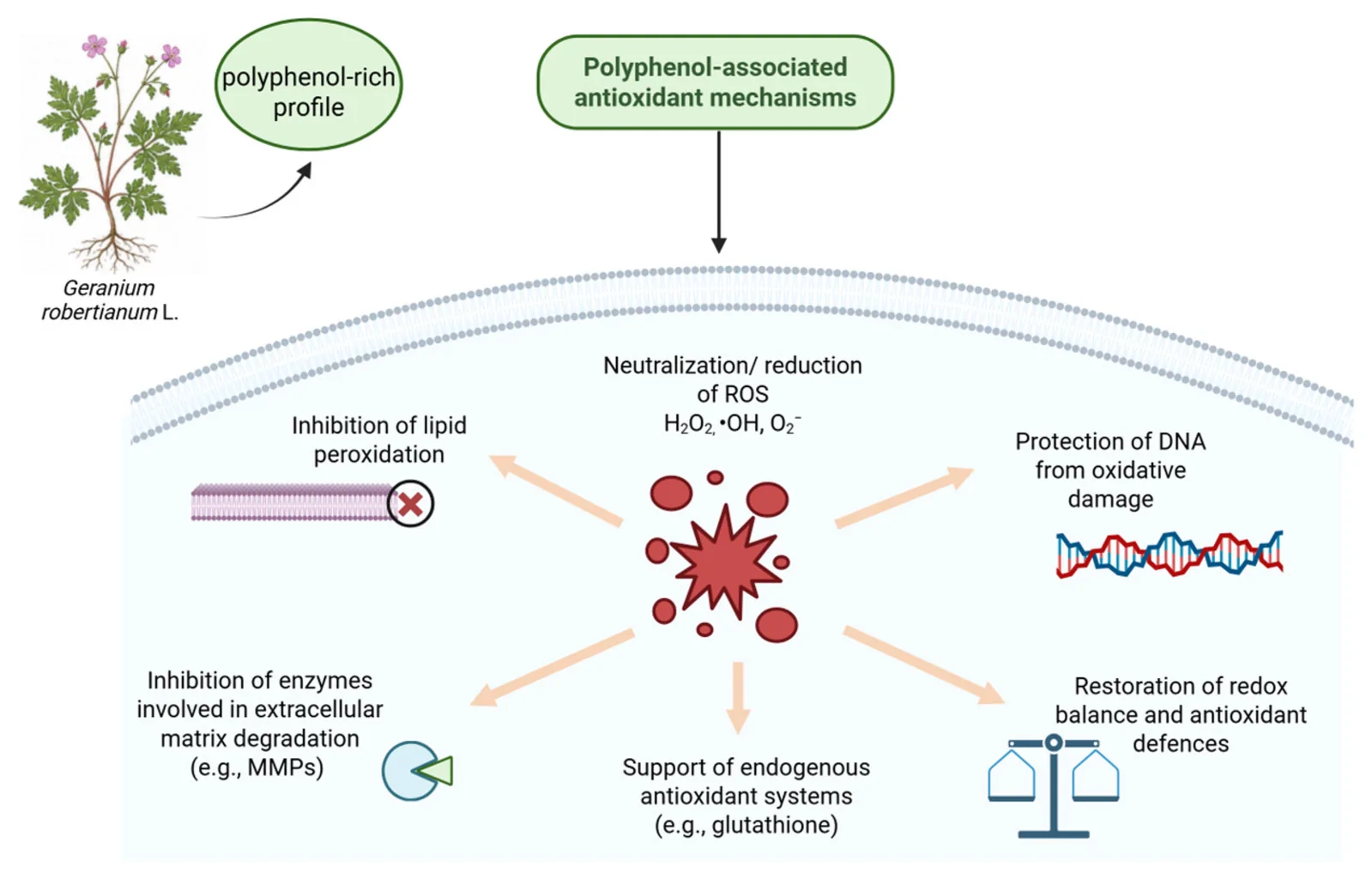
Antioxidant mechanisms associated with a high polyphenolic content. Created with BioRender.com.

The antioxidant potential of GR is strongly related to its polyphenolic profile, which enables the neutralization of ROS and the limitation of lipid peroxidation and DNA damage (Figure 6) [8,39]. Although these antioxidant effects have not yet been directly investigated in oral disease models, they suggest a potential relevance of GR extracts to oxidative imbalance involved in periodontal inflammation, caries progression, mucosal damage, and oral carcinogenesis [76,77,78].

### 5.6. Wound-Healing and Hemostatic Activity

GR has been traditionally used for wound care and haemostatic purposes, including the topical treatment of cuts, persistent ulcers, throat irritation, and inflammatory lesions. In the mountain regions of Montenegro, infusions prepared from leafy stems have been used for poorly healing ulcers, supporting the ethnomedicinal relevance of this species in tissue repair [8,9]. These traditional applications are of interest for oral medicine, where rapid mucosal healing and control of local bleeding are important after traumatic lesions, ulcerations, surgical procedures, and inflammatory mucosal conditions [80].

The potential relevance of GR to wound healing may be associated with geraniin and ellagic acid, two compounds with documented roles in tissue regeneration [8,9]. Geraniin has been reported to modulate inflammatory pathways involved in tissue repair by lowering TNF-α and NF-κB activity, mechanisms that are relevant during the early inflammatory phase of wound healing [81]. Furthermore, in vivo studies using excision and incision wound models showed that geraniin significantly enhanced wound contraction and hydroxyproline levels, indicating enhanced collagen synthesis. Histological and immunohistochemical analyses also showed elevated expression of transforming growth factor beta 1 (TGF-β1) in treated tissues, a key regulator of fibroblast proliferation, collagen deposition, angiogenesis, and tissue contraction. Additionally, geraniin treatment significantly improved tensile strength in incision wounds, suggesting improved collagen architecture and tissue regeneration [82].

Ellagic acid, also identified in GR extracts, provides evidence supporting the possible relevance of GR to oral wound healing. Research by Tekin et al. showed that applying ellagic acid to gingival injuries in rats significantly improved epithelial regeneration, boosted collagen fiber formation, and increased levels of fibroblast growth factor (FGF) and epidermal growth factor (EGF), critical growth factors in tissue repair, supporting its role as a promising agent for enhancing oral healing [80]. At the cellular level, ellagic acid enhanced fibroblast viability and migration while stimulating the expression of fibronectin and α-actin [83]. Under inflammatory conditions, it also promoted fibroblast proliferation, while suppressing key pro-inflammatory cytokines, IL-1β and IL-6, promoting a favorable environment for tissue recovery [84].

The hemostatic effect of GR is linked to its high tannin content. Ellagitannins release ellagic acid after hydrolysis, which interacts with metal cations such as magnesium and zinc and forms insoluble complexes involved in coagulation-related processes [85].

**Figure 7:**
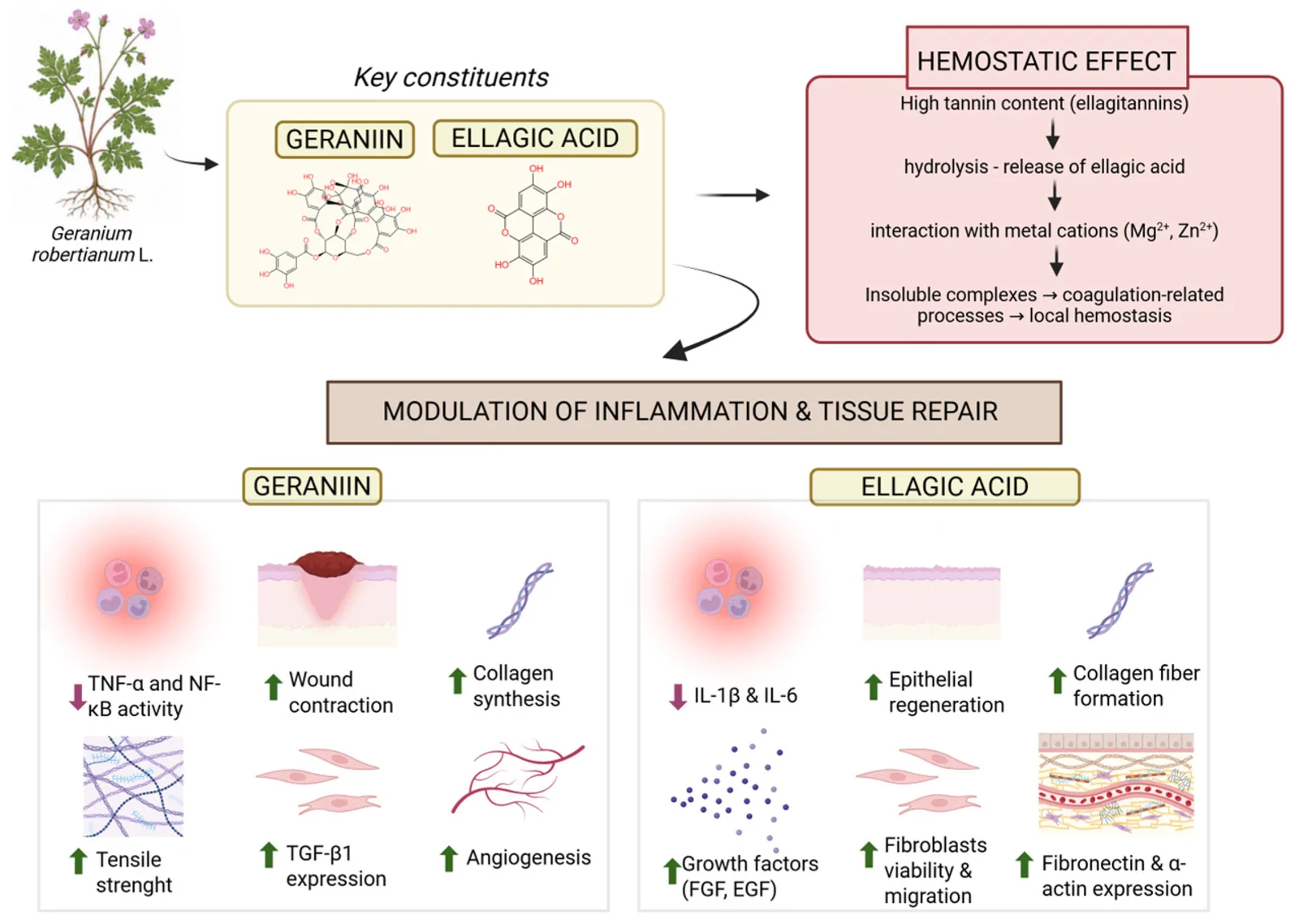
Wound healing and hemostatic effects reported for bioactive constituents also found in GR. Created with BioRender.com.

The presence of geraniin, ellagic acid, and other tannins in GR supports its potential relevance to wound healing and hemostasis. Studies on these individual constituents indicate that they may modulate key processes involved in oral tissue repair, including inflammation control, fibroblast activity, collagen synthesis, epithelial regeneration, and local hemostasis (Figure 7). However, the potential application of GR in oral wound management requires further investigation.

### 5.7. Anticancer Effects in Oral Cancer

Oral cancer is frequently diagnosed at advanced stages and is associated with high recurrence rates and limited survival, despite progress in chemotherapy and radiotherapy. Conventional therapies are often accompanied by significant side effects, which has encouraged the investigation of plant-derived compounds as complementary sources of anticancer molecules [86,87]. GR has shown cytotoxic activity in vitro against several cancer cell lines, including those derived from head and neck, cervical, laryngeal, colorectal, hepatic, and lung cancers. Its phytochemical composition provides a biochemical basis for investigating its anticancer potential in oral malignancies [43]. The most relevant evidence for oral oncology comes from a study using A253 human salivary gland tumor cells. The study showed that GR ethanolic extract (Timisoara, Romania) reduced cancer cell proliferation and colony-forming ability. Moreover, GR treatment also increased intracellular ROS and activated caspase-3/7 and caspase-9, indicating induction of apoptosis through the mitochondrial pathway. Thus, GR extract affected malignant salivary gland cells by combining oxidative stress modulation with activation of apoptotic mechanisms [43].

**Figure 8:**
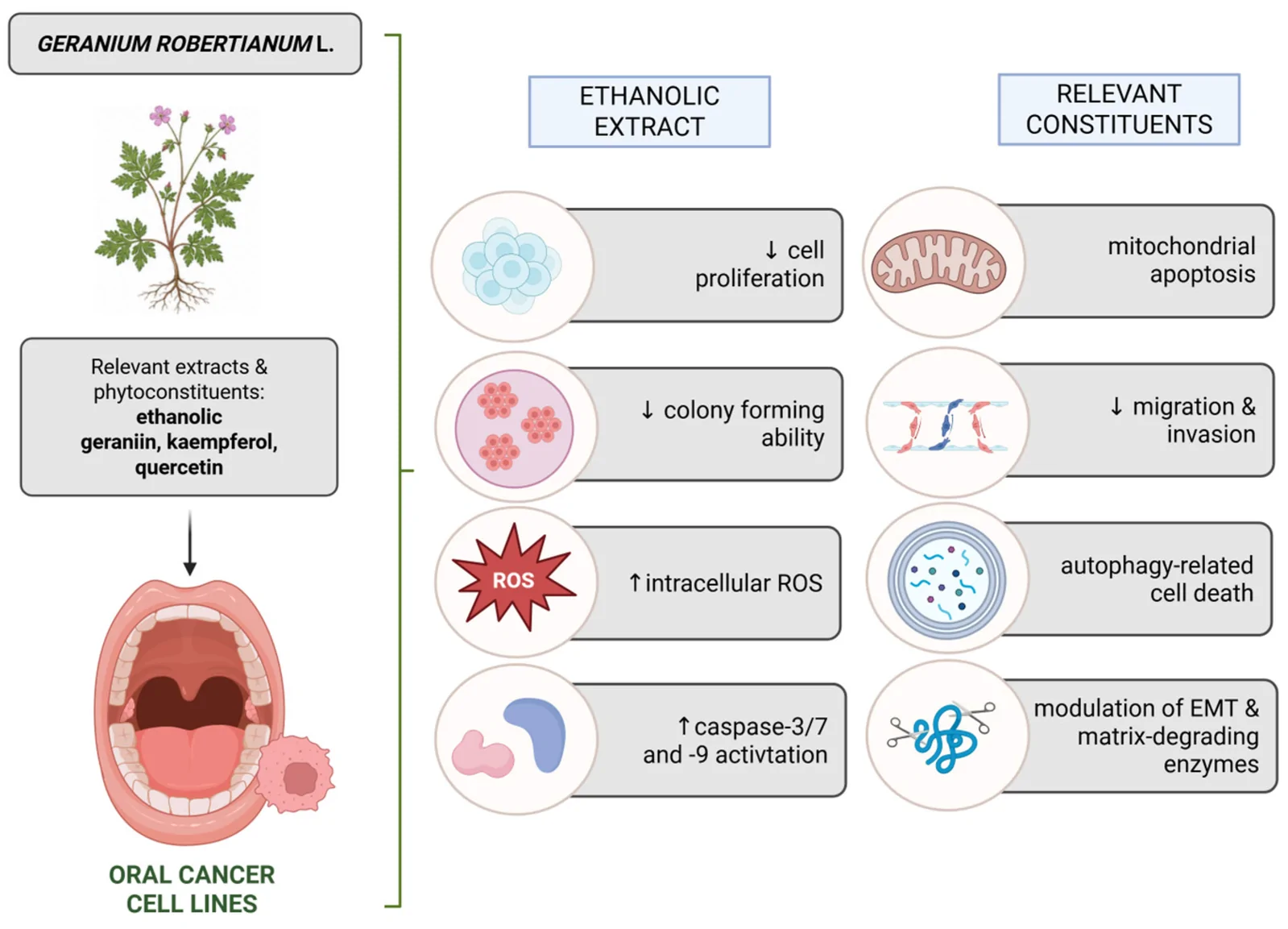
Anticancer mechanisms of GR and bioactive constituents also found in the plant, relevant to oral cancer. Created with BioRender.com.

Several compounds identified in GR further support its anticancer relevance. Geraniin has been reported to inhibit proliferation and induce autophagy-related cell death in nasopharyngeal carcinoma cells [47]. Kaempferol has demonstrated activity against MC-3 human oral cancer cells by inducing apoptosis through mitogen-activated protein kinase (MAPK)-related pathways and activating autophagy through c-Jun N-terminal kinase (JNK) signalling. These effects were also supported by in vivo findings in animal models [87]. Quercetin has been investigated in several studies on oral squamous cell carcinoma and has been shown to inhibit cancer cell proliferation, migration, and invasion, while showing lower toxicity toward normal oral epithelial cells. Its anticancer properties are linked to the modulation of multiple pathways involved in tumour progression, including epithelial–mesenchymal transition and matrix-degrading enzymes [86,88,89]. These findings position GR as a promising source of bioactive compounds with adjunctive potential in oral oncology (Figure 8).

**Figure 9:**
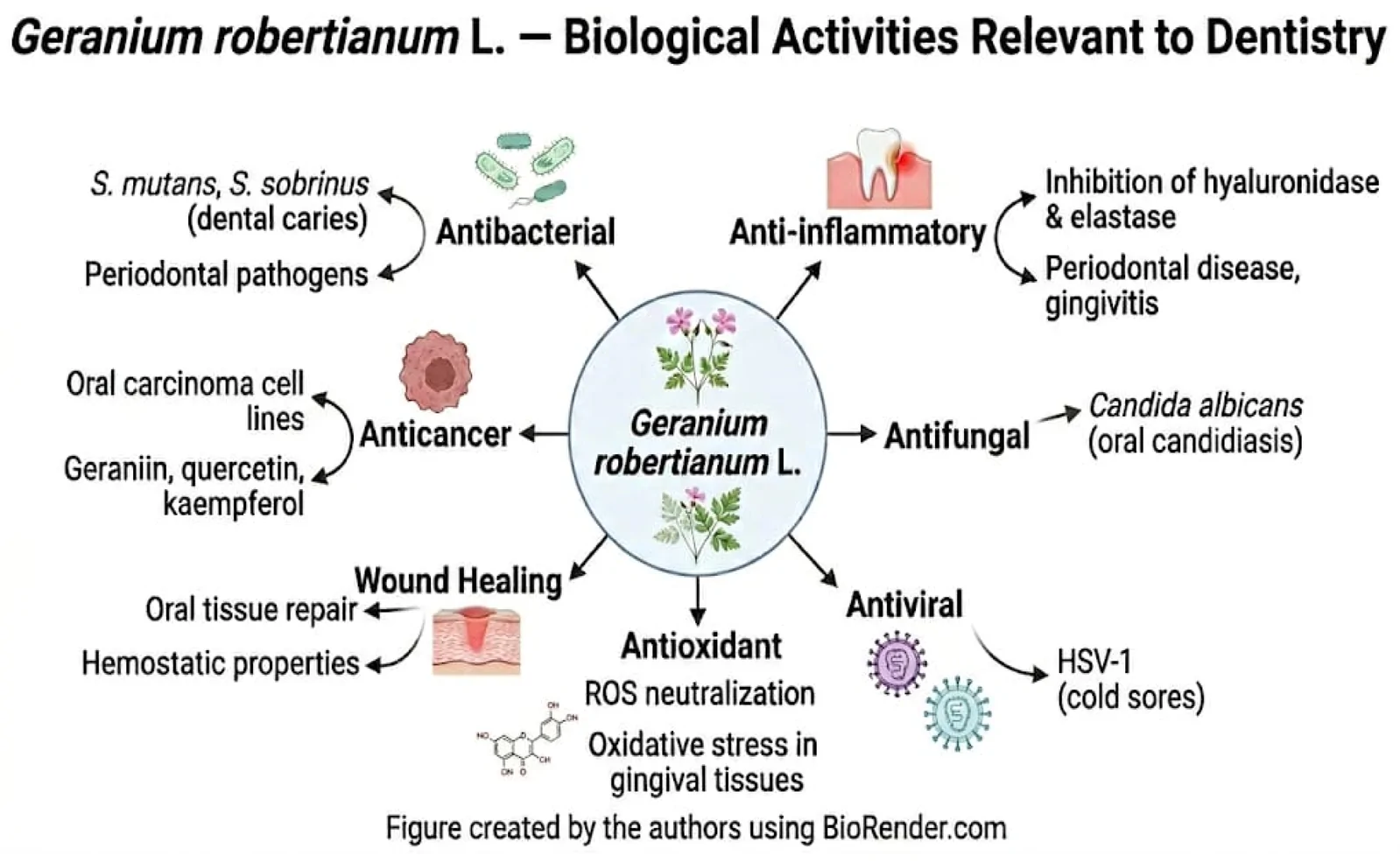
Biological activities of *Geranium robertianum* L. relevant to dentistry.

**Figure 10:**
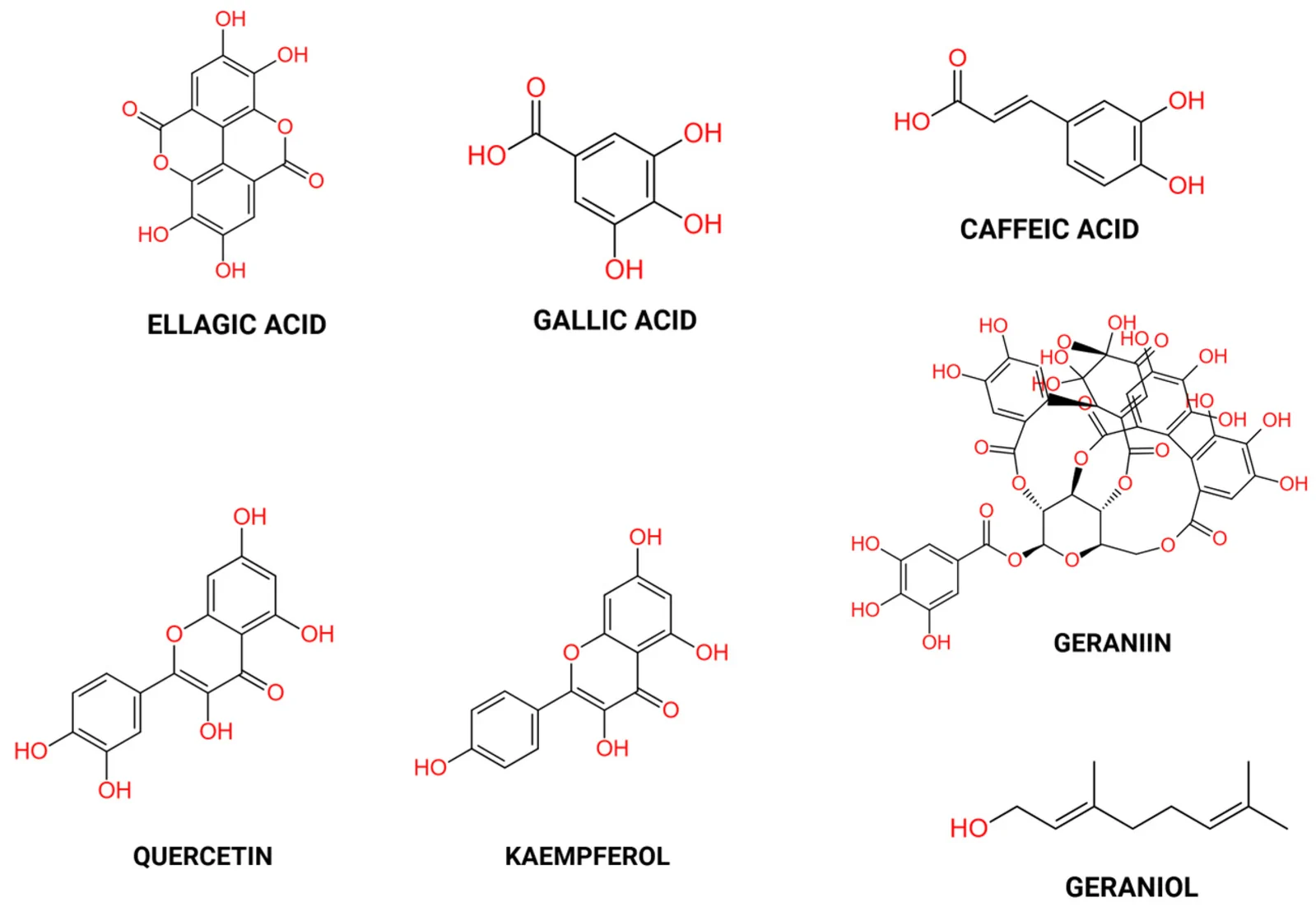
Illustration of the most bioactive compounds found in GR.

The main biological activities of *Geranium robertianum* L., with potential relevance to dentistry, are summarized in Figure 9, including antibacterial, antifungal, antiviral, anti-inflammatory, antioxidant, wound-healing, and anticancer effects. Moreover, Figure 10 illustrates the most bioactive compounds found in GR.

A comprehensive overview of the reported biological activities of *Geranium robertianum* L., the associated phytochemical constituents, experimental models employed, and the corresponding levels of confidence is provided in Table 3.

## 6. Comparative Evaluation of GR and Other Standardized Extracts Used to Manage Oral Diseases

In order to evaluate the therapeutic potential of GR within contemporary oral phytotherapy, the review also aimed to compare the described data from the literature with well-established plant-derived standardized extracts that have already been used in the management of oral diseases, such as sage (*Salvia officinalis* L.), turmeric (*Curcuma longa* L.), chamomile (*Matricaria chamomilla* L.), and marigold (*Calendula officinalis* L.). Extracts obtained from these species are generally associated with a predominant therapeutic action, meanwhile GR appears to combine several of these actions within a single phytocomplex.

Different types of extracts from *Salvia officinalis* L. are traditionally valued for their antiseptic and astringent properties, attributed to rosmarinic acid and its essential oil constituents. In a recent twelve-week randomized controlled trial, a 2% sage aqueous infusion reduced dental plaque and gingival inflammation in young adults. Its efficacy was similar to chamomile and ginger-based rinses, and it demonstrated a better tolerability profile than chlorhexidine, without staining or taste alteration. The authors emphasized that without mechanistic assays, these clinical improvements cannot be interpreted as direct anti-inflammatory [90]. GR shares this astringent, tannin-based mode of action but, in contrast to sage, its antibacterial activity against cariogenic streptococci (*S. mutans*, *S. sobrinus*) has so far been demonstrated only in vitro [9].

Curcumin, the main phytochemical from *Curcuma longa* L., is recognized mainly as an anti-inflammatory agent. A systematic review of clinical trials found that curcumin, used as an adjunct to oral hygiene, significantly reduced plaque, gingival, and bleeding indices, with an effect similar to that of chlorhexidine [91]. A comparable anti-inflammatory mechanism has been attributed to GR, whose ellagitannins, ellagic acid, gallic acid, and quercetin modulate NF-κB signaling and pro-inflammatory cytokine release, although the corresponding evidence remains preclinical [8,55].

Different types of extracts from *Matricaria chamomilla* L. are mainly used as soothing, anti-inflammatory agents for irritated oral mucosa. A systematic review of randomized trials reported that topical chamomile extract (1–2.5%) was effective in the prevention and/or treatment of radio-chemotherapy-induced oral mucositis [92]. GR is likewise rich in anti-inflammatory flavonoids and has documented mucosal wound-healing potential, yet its use in mucositis or symptomatic mucosal conditions has not been clinically evaluated [8].

Different types of extracts from *Calendula officinalis* L. are best known as cicatrizing agents. In a randomized clinical study including 240 patients, a calendula mouthwash significantly reduced plaque, gingival, and bleeding indices, supported by its recognized wound-healing, anti-inflammatory, and antimicrobial activities [93]. A similar profile underlies the wound-healing and hemostatic actions attributed to GR through geraniin, ellagic acid, and its high tannin content, which promote collagen synthesis, epithelial regeneration, and local hemostasis [8,9].

Taken together, these comparisons suggest that the distinctive feature of GR is not represented by a single dominant property, but the combination of antibacterial, anti-inflammatory, antioxidant, wound-healing, and hemostatic actions assigned to polyphenol- and tannin-rich phyto-complexes. Such actions are typically provided individually by other phytotherapeutic agents [6,55]. The main difference, however, lies in the level of evidence: sage, curcumin, chamomile, and calendula are already supported by human clinical trials in defined oral conditions, whereas the oral-health evidence for GR remains almost exclusively in vitro and preclinical [8,9,43]. This comparison reinforces the view that GR is a promising candidate, whose rich phytochemical profile justifies dedicated clinical investigation in dentistry.

## 7. Current Evidence, Clinical Translation, and Future Perspectives

The available evidence presents both strengths and limitations. The phytochemical composition of GR has been extensively characterized, and several studies report antimicrobial, antioxidant, anti-inflammatory, wound-healing, and cytotoxic activities assigned to its extracts or major constituents. In addition, part of the biologically active compounds were evaluated in experimental models relevant to oral diseases, thus providing preliminary support for the potential application of GR in oral health. On the other hand, most studies investigating GR remain limited to in vitro assays and preclinical models not specifically related to oral diseases, while part of the evidence derives from studies on individual bioactive constituents identified in GR and cannot be extrapolated to the plant extract. The heterogeneity of extraction methods, tested concentrations, and experimental models further limits comparison between studies. Therefore, the available findings support the potential relevance of GR in the management of oral diseases. However, this evidence needs to be strengthened and confirmed through clinical and disease-specific experimental models.

The clinical translation of GR remains limited by several important challenges. Differences in extraction methods and phytochemical composition may affect the reproducibility, stability, bioavailability, and efficacy of plant-derived preparations. Furthermore, additional investigation is required to establish standardized formulations, effective dosage, long-term safety, potential toxicity, interactions with conventional treatments, and the regulatory requirements for clinical use [94].

Safety, bioavailability, and pharmacokinetic data for GR are limited. Isolated cases of contact sensitization and allergic contact dermatitis have been reported, suggesting a potential hypersensitivity risk in susceptible individuals [95,96]. However, their relevance to oral mucosal exposure remains unknown. Moreover, the absorption, distribution, metabolism, or elimination of GR extracts have not been studied. On the other hand, research on geraniin and ellagic acid shows that these compounds present limited and variable intestinal bioavailability [97,98]. Therefore, the bioavailability, pharmacokinetic profile, toxicity, and local oral tolerability of GR extracts require further investigation.

This review provides the scientific community with a comprehensive overview of the evidence currently available in the literature, bringing together the main findings reported to date, as well as highlighting the existing gaps. By offering an up-to-date synthesis of the published data, the review provides an important foundation for guiding future research. Nevertheless, further research directions should focus on the development and standardization of GR extracts, followed by mechanistic investigation in oral disease-specific preclinical models. In this regard, dose–response relationships, bioavailability, local and systemic safety, and compatibility with conventional oral therapies should also be evaluated. Furthermore, clinical trials should be conducted using standardized GR-based oral healthcare products.

## 8. Conclusions

*Geranium robertianum* L. emerges as a promising phytotherapeutic candidate for oral health due to its rich phytochemical profile, particularly its content of hydrolysable tannins, flavonoids, and phenolic acids. The available evidence suggests that geraniin, ellagic acid, gallic acid, quercetin, and kaempferol may contribute to several biological activities relevant to dentistry, including antimicrobial, anti-inflammatory, antioxidant, wound-healing, and anticancer effects. These properties support the potential use of GR as an adjunctive natural approach in the prevention and management of oral diseases. Nevertheless, current evidence remains largely preclinical, and further studies are needed before its efficacy and safety can be confirmed for dental practice.

## Figures and Tables

**Table 1:** Taxonomic classification of *Geranium robertianum* L., according to Plants of the World Online, Royal Botanic Gardens, Kew [14].

Taxonomic Rank	Taxon
Kingdom	Plantae
Phylum	Streptophyta
Class	Equisetopsida
Subclass	Magnoliidae
Order	Geraniales
Family	Geraniaceae
Genus	*Geranium*
Species	*Geranium robertianum*

**Table 2:** The total content of polyphenols, flavonoids, and the main bioactive compounds of GR plant extracts.

Plant Material	Extraction Solvent/Method	Plant Source	TPC	TFC	Predominant Phenolic and Flavonoid Compounds	Reference
Leaves	Aqueous (8% mass)	Giza, Egypt	194.96 mg GAE/g	-	Chlorogenic acid: 443.84 µg/g Gallic acid: 146.87 µg/g Ellagic acid: 115.65 µg/g Rutin: 230.84 µg/g	[22]
Leaves	Decoction	Cernache do Bonjardim, Portugal	649.2 mg GAE/g	-	Chlorogenic acid: 14.9 mg/g	[31]
Stems			536.4 mg GAE/g	-	Chlorogenic acid: 19.6 mg/g	
Leaves	6% aqueous	Unspecified	1.96 mg/mL	-	-	[30]
	10% aqueous		3.19 mg/mL	-	-	
	Ethanolic 50% (10% mass concentration)		4.21 mg/mL	-	-	
	Ethanolic 70% (10% mass concentration)		3.38 mg/mL	-	-	
Leaves	Aqueous, microfiltrated	Romania	Almost 700 mg GAE/L	Almost 100 mg rutin/L	Caffeic acid: 8.18 mg/kg Ferulic acid: 1.18 mg/kg *p*-Coumaric acid: 0.77 mg/kg Rutin: 2.67 mg/kg Quercetin: 4.83 mg/kg	[37]
	Ethanol 50%, microfiltrated		Over 800 mg GAE/L	Over 150 mg rutin/L	Caffeic acid: 20.18 mg/kg Ferulic acid: 11 mg/kg *p*-Coumaric acid: 9.22 mg/kg Rutin: 38.95 mg/kg Quercetin: 54.6 mg/kg	
Flowers	Acetone	Manjača, Bosnia and Herzegovina	-	4.2%	-	[38]
Whole plant	Infusion	Trás-os-Montes, Portugal	228 mg GAE/g	35.9 (mg CE/g)	-	[26]
	Decoction		212 mg GAE/g	34.57 (mg CE/g)		
	n-hexane		30.7 mg GAE/g	-		
	Dichloromethane		3.8 mg GAE/g	-		
	Ethyl acetate		176 mg GAE/g	50 (mg CE/g)		
	Acetone		347 mg GAE/g	53 (mg CE/g)		
	Methanol		268 mg GAE/g	48 (mg CE/g)		
Aerial parts	Methanol (drug/solvent ratio 1:10)	Vlasina plateau, Serbia	425.31 mg GAE/g	-	Tannin content: 270.72 mg GAE/g	[39]
Underground parts			390.29 mg GAE/g	-	Tannin content: 233 mg GAE/g	
Aerial parts	Ethanol 80%	Samobor, Croatia	119.6 mg GAE/g	26.7 mg GAE/g	Gallic acid: 0.2 mg/g Catechin: 0.45 mg/g Quercetin-4-glucoside: 0.81 mg/g Rutin: 2.13 mg/g	[40]
Unspecified	Aqueous 6%, microfiltrated	Unspecified	-	-	Gallic acid: 978.86 µg/g vegetal mass Ellagic acid: 744.15 µg/g vegetal mass Luteolin: 3.56 µg/g vegetal mass Rutin: 1.73 µg/g vegetal mass	[11]
	Aqueous 6%, ultrafiltrated Millipore 10.000 Da (permeate)		675.04 µg/g	-	-	
	Aqueous 6%, ultrafiltrated Millipore 10.000 Da (concentrate)		745.61 µg/g	-	Gallic acid: 1056.79 µg/g vegetal mass Ellagic acid: 779.63 µg/g vegetal mass Luteolin: 4.03 µg/g vegetal mass Rutin: 3.83 µg/g vegetal mass	
Aerial parts	Aqueous lyophilized	Warsaw, Poland	26.9 g/100 g	-	Total tannin content: 20.7%	[41]
Aerial parts	Ethanol 70%	Baile Herculane, Romania	140.14 mg GAE/g	5.97 mg QE/g	-	[42]
Aerial parts	Ethanol 70%	Timisoara, Romania	191.7 mg GAE/g	117.4 mg QE/g	-	[43]

TPC = total polyphenolic content; TFC = total flavonoid content; GAE = gallic acid equivalents; CE = catechin equivalents; QE = quercetin equivalents.

**Table 3:** Summary of the reported biological activities of *Geranium robertianum* L. extracts or active phytocompounds relevant to dental medicine.

Biological Activity	Corresponding Phytochemical Constituents	Experimental Model	Level of Evidence ^a^	Ref.
Antibacterial activity	Polyphenols of the GR aqueous extract (aerial parts)	In vitro: Gram-positive and Gram-negative bacteria	++	[9]
	Polyphenols of the GR dichloromethane extract (leaves)	In vitro: *Staphylococcus aureus* (inhibition zone 8 mm at 1 mg/mL)	++	[12]
	Polyphenols of the GR ethanolic extract (aerial parts)	In vitro: Gram-positive and Gram-negative bacteria (inhibition comparable to levofloxacin)	++	[43]
Antifungal effect in oral candidiasis	Polyphenols of GR ethyl acetate, aqueous and hexane extracts (aerial parts)	In vitro: *Candida* spp., including *C. albicans* and non-*albicans* species	+++	[9]
	Ellagic acid (as an ellagic acid/HP-β-CD complex) ^b^	In vitro and in vivo: *C. albicans*, oral candidiasis model	++	[63]
	Geraniol ^b^	In vitro: *Candida* biofilm and oral epithelial cell inflammatory model	++	[64]
Anti-inflammatory activity in periodontal disease	Gallic acid ^b^	In vitro: periodontal ligament stem cells from stage III periodontitis patients	++	[59]
	Quercetin ^b^	In vitro: human gingival fibroblasts stimulated with *Porphyromonas gingivalis* LPS	++	[56]
	Ellagic acid ^b^	In vivo: experimental periodontitis in rats	++	[58]
Antiviral activity in oral viral infections	Geraniin ^b^	In vitro: HSV-1 plaque reduction assay (Vero cells)	++	[69]
	Quercetin ^b,c^	Review reporting a clinical trial in 68 patients with oral herpes	+	[73]
Antioxidant activity	Methanolic GR extracts; geraniin	In vitro: DPPH radical-scavenging and β-carotene bleaching assays	++	[12,15,39]
Anticancer activity	Ethanolic GR extract (aerial parts)	In vitro: A253 human salivary gland carcinoma cells (reactive oxygen species, apoptosis)	+++	[43]
	Geraniin ^b^	In vitro: nasopharyngeal carcinoma C666-1 cells	+	[47]
	Kaempferol ^b^	In vitro and in vivo: MC-3 human oral cancer cells (xenograft)	++	[87]
	Quercetin ^b^	In vitro: oral squamous cell carcinoma cell lines	++	[88]
Wound-healing and haemostatic activity	Ellagic acid ^b^	In vivo: gingival wound model in rats	++	[80]
	Geraniin ^b^	In vitro: HaCaT keratinocytes (skin)	+	[81]
	Geraniin ^b^	In vivo: rat excision and incision wound models (skin)	+	[82]
	Hydrolysable tannins (ellagic acid)	In vitro: coagulation-related processes (metal-cation complexation)	+	[85]

^a^ Level of evidence, assigned according to the relevance of the experimental model to the oral cavity: +++ GR extracts tested in models of direct oral relevance (oral pathogens, oral or salivary gland cells); ++ GR extracts tested in models of indirect oral relevance, or constituents identified in GR tested in oral-relevant models; + constituents identified in GR tested exclusively in non-oral models. ^b^ The constituent was identified in the phytochemical profile of GR but, in the study cited, was isolated from another plant species; no study has evaluated it purified from GR itself. ^c^ The trial evaluated a multi-ingredient herbal preparation containing 100 mg quercetin, not quercetin as a single agent.

## Data Availability

The original contributions presented in this study are included in the article. Further inquiries can be directed to the corresponding author.

## Abbreviations

The following abbreviations are used in this manuscript:

ATCC	American Type Culture Collection
CE	Catechin Equivalents
DNA	Deoxyribonucleic Acid
DPPH	2,2-Diphenyl-1-Picrylhydrazyl
EGF	Epidermal Growth Factor
FGF	Fibroblast Growth Factor
GAE	Gallic Acid Equivalents
GR	*Geranium robertianum* L.
HIV	Human Immunodeficiency Virus
HP-β-CD	Hydroxypropyl beta-cyclodextrin
HRV	Human Rhinovirus
HSV	Herpes Simplex Virus
IC50	Half maximal inhibitory concentration
IL-1β	Interleukin-1 beta
IL-6	Interleukin-6
IL-8	Interleukin-8
IL-10	Interleukin-10
IL-18	Interleukin-18
JNK	c-Jun N-terminal Kinase
LPS	Lipopolysaccharide
MAPK	Mitogen-Activated Protein Kinase
MBC	Minimum Bactericidal Concentration
MFC	Minimum Fungicidal Concentration
MIC	Minimum Inhibitory Concentration
MMP-8	Matrix Metalloproteinase-8
MMP-9	Matrix Metalloproteinase-9
NF-κB	Nuclear Factor kappa-light-chain-enhancer of activated B cells
ORL	Otorhinolaryngology
PGE2	Prostaglandin E2
PI3K/Akt	Phosphoinositide 3-Kinase/Protein Kinase
*PLB1*	Phospholipase B1
PPARγ	Peroxisome Proliferator-Activated Receptor gamma
QE	Quercetin Equivalents
*SAP1*	Secreted Aspartyl Proteinase 1
SARS-CoV-2	Severe Acute Respiratory Syndrome Coronavirus 2
TFC	Total Flavonoid Content
TGF-β1	Transforming Growth Factor beta 1
TLR-4	Toll-Like Receptor 4
TNF-α	Tumor Necrosis Factor alpha
TPC	Total Polyphenolic Content
WHO	World Health Organization
